# Hidden in Plain Sight: Neglected Congenital Ranula Presenting as Failure to Thrive in an Infant

**DOI:** 10.7759/cureus.89710

**Published:** 2025-08-09

**Authors:** Soumi Kundu, Medhagopal R. G, Sarthak Das, Saroj K Tripathy, Md. Ehtesham Ansari

**Affiliations:** 1 Pediatrics, All India Institute of Medical Sciences Deoghar, Jharkhand, IND

**Keywords:** congenital ranula, failure to thrive, marsupialization, neonatal oral cyst, pediatric feeding difficulty

## Abstract

Congenital ranula is a rare mucous extravasation cyst arising from the sublingual gland, presenting early in life and potentially causing feeding difficulties and growth failure. We report a two-month-old infant with a large intraoral swelling present since birth, leading to poor weight gain and tongue displacement. Ultrasound confirmed a plunging ranula, and marsupialization was successfully performed. Post-surgery, the infant showed significant improvement in feeding and growth. This case highlights the importance of early oral cavity examination in neonates, as timely identification and intervention can prevent complications and ensure improved nutritional and developmental outcomes in affected infants.

## Introduction

Ranula is a mucous-filled cystic lesion arising from the sublingual gland due to ductal obstruction or extravasation of mucus into adjacent tissues [1]. It can be either congenital or acquired and is classified into different types based on the anatomical location and extension. The incidence rate of ranula is 0.02% per 1000 individuals [2]. Sometimes, they remain undetected until they progress to a size that causes respiratory difficulties, feeding issues, or other problems. To avoid these consequences, early diagnosis and treatment are essential.

Congenital ranulas are rare, often presenting as asymptomatic swellings in the floor of the mouth, but they may become symptomatic as they enlarge. Their subtle presentation in neonates and infants can delay diagnosis, especially in resource-limited settings. Prompt recognition is vital, as untreated lesions may interfere with oral intake, leading to nutritional compromise and failure to thrive.

We report a case of a congenital ranula causing failure to thrive in an infant.

## Case presentation

**Figure 1:**
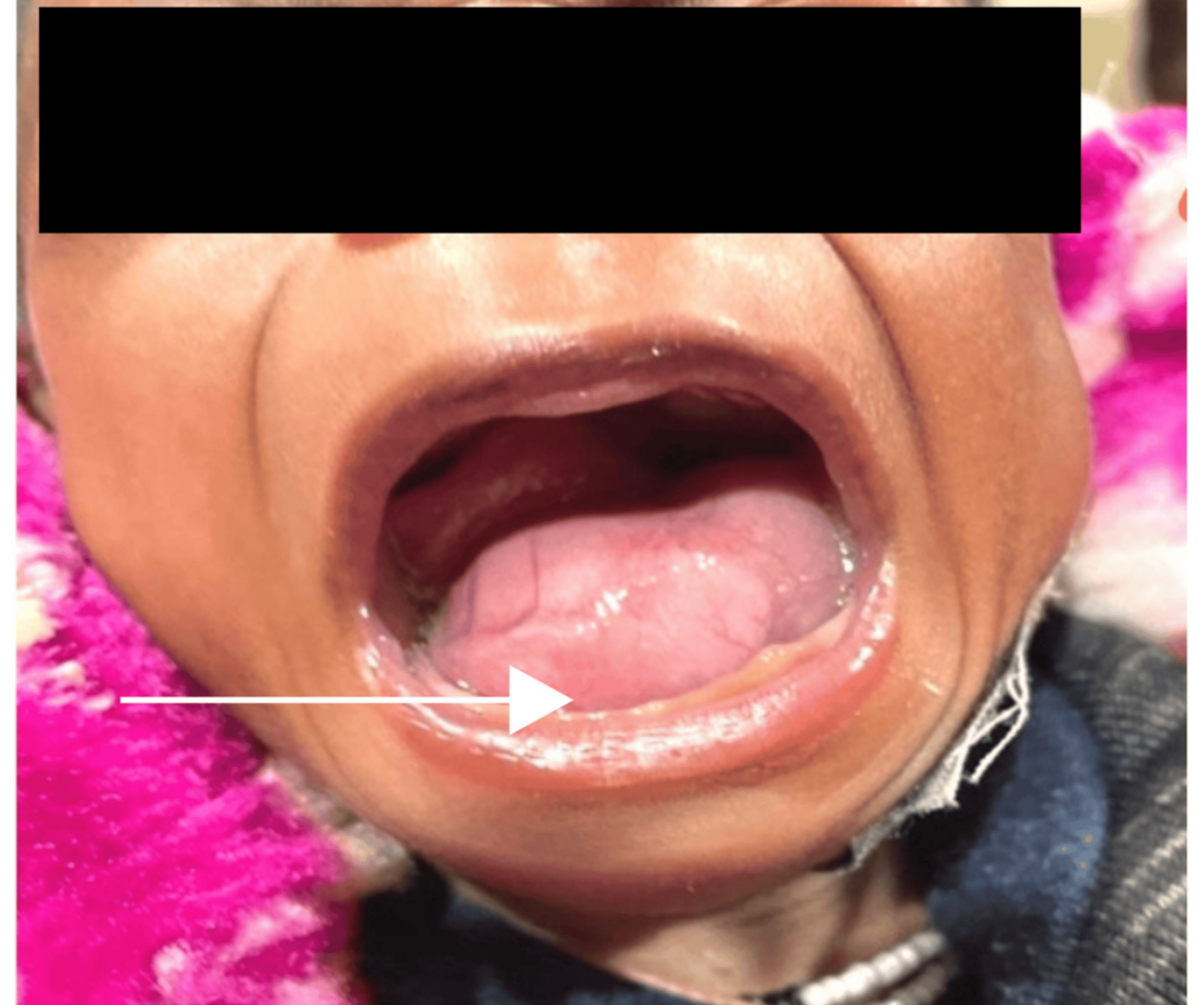
Swelling on the floor of the mouth (pre-operative) showing the pinkish cystic lesion on the floor of the mouth resembling a “frog belly”

A two-month-old term boy, delivered by normal vaginal delivery at the hospital with a birth weight of ~3 kg, weight-for-height and height-for-age between 0 and + 1 standard deviations (SD), was brought to the OPD with complaints of swelling on the floor of the mouth (Figure 1), noticed since birth. There was no history suggestive of trauma or infection. The swelling was gradually progressive in size, causing difficulty in feeding and the tongue to fall back. Occasionally, the mother noticed noisy breathing. The antenatal period was uneventful with a normal amniotic fluid index (AFI). The mother was a booked case and had done all antenatal checkups, and all trimester scans were normal.

On day 3 of life, his grandmother first noticed the swelling, which later led to disturbance in direct breastfeeding. Still, the mother continued direct breastfeeds. Consequently, the child was not gaining weight and had an inconsolable cry, which made them seek our medical facility.

**Figure 2:**
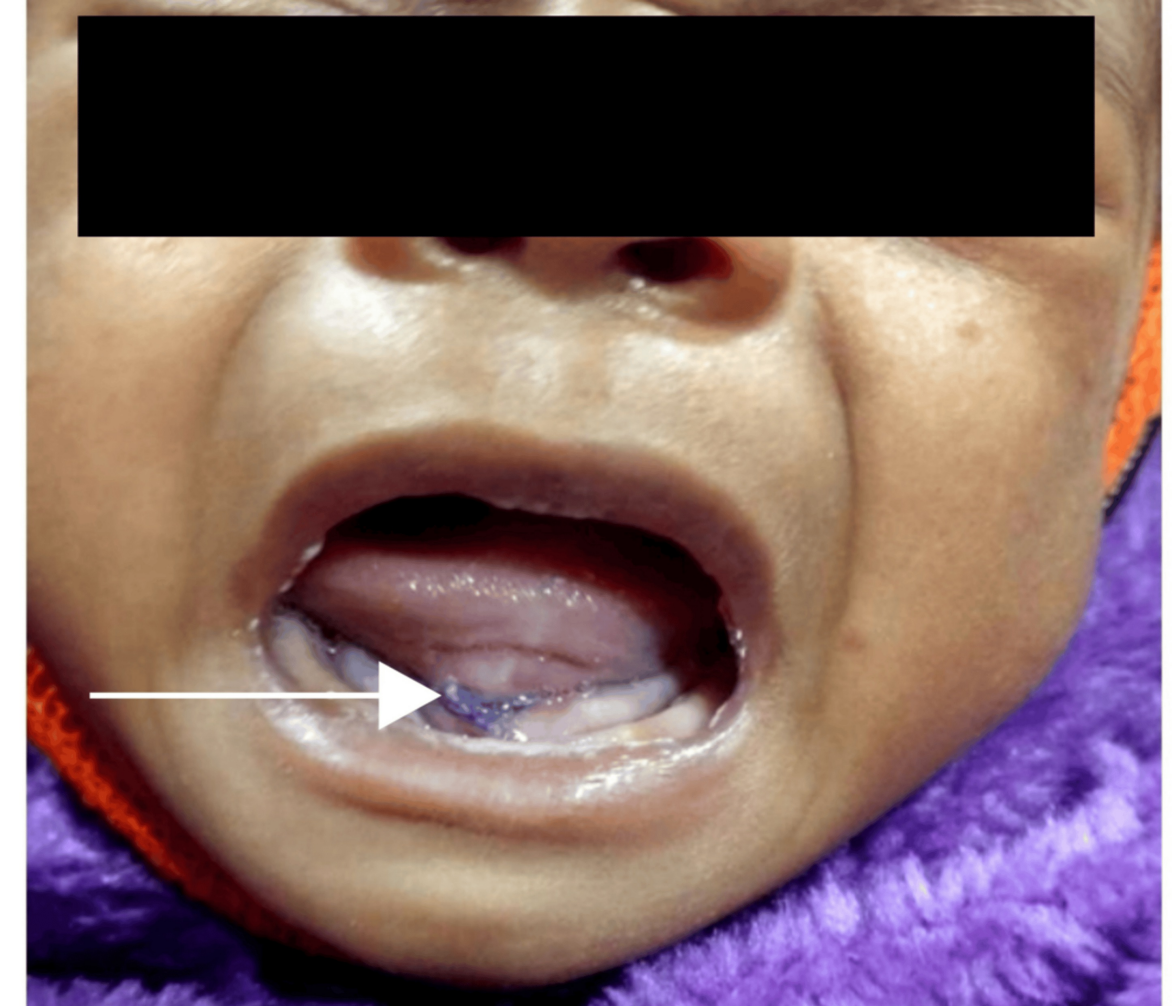
There is no swelling on the floor of the mouth or tongue fall-back after marsupialization (postoperative) The tongue base has been sutured together and to the floor of the mouth.

On examination, the child was thin-built and malnourished, with no dysmorphic facies. Anthropometric assessment revealed that both weight-for-height and height-for-age were between -3 and -2 SD, while head circumference for age was below -3 SD. Vital signs were stable. Systemic examination was normal. Heart sounds were heard, and there was no murmur. Bilateral air entry was present with vesicular breath sounds, but there were no added sounds. On GI system examination, the abdomen was scaphoid in shape, soft, non-tender, with no organomegaly. Local oral examination showed a 144x3 cm pinkish swelling, cystic in consistency, fluctuant and soft in palpation, but with no warmth/tenderness on the floor of the mouth, with noticeable tongue fall-back. We suspected the possibilities of a ranula or a dermoid or thyroglossal cyst. Blood investigations were sent, which revealed normal blood counts, liver function test (LFT), renal function test (RFT), and electrolytes. Peripheral smear showed no atypical cells. CRP was negative. An urgent ultrasound was performed, which showed features suggestive of a left plunging ranula. A pediatric surgical consultation was sought, and aspiration of the lesion yielded dirty white content. The child was then admitted to our hospital on January 28, 2025, at the age of two months, and marsupialization was performed the next day (Figure 2) [3]. The specimen, along with the dirty white aspirate obtained, was sent for histopathological examination. The gauge was kept inside the oral cavity for one day and removed the next day, following which oral feeding was started.

Postoperatively, the child tolerated direct breastfeeding well. On follow-up after two weeks (at 2.5 months), the child demonstrated satisfactory weight gain of ~300 grams (= 20 to 25 grams/day) and was thriving well.

## Discussion

The term ‘ranula’ stems from the Latin word 'Rana,' which means frog, due to its resemblance to a frog's underbelly [3]. Hippocrates attributed ranula to chronic inflammation, but Paré believed that it signifies the descent of the brain and pituitary matter (Historical). Boyd et al. described a ranula as a dilatation of the submandibular gland duct [4]. A ranula results from the obstruction of the sublingual gland ducts, leading to proximal expansion, or by disruption of minor salivary ducts that cause mucus to extravasate in surrounding structures, which lack an epithelial lining [5].

Lazzeroni et al. classified ranulas into three types: Type 1 - Intraoral; Type 2 - Cervical; Type 3 - Extended [6]. Each type is further subclassified into "a" and "b" categories: Type 1a is a simple endoral unilateral sublingual ranula; Type 1b is a simple endoral sublingual ranula that extends to the contralateral oral floor; Type 2a is a sublingual plunging ranula that extends from a mylohyoid muscle hiatus to the cervical region; Type 2b is a sublingual plunging ranula that extends from the posterior margin of the mylohyoid muscle to the cervical region; Type 3a is an extended sublingual ranula that involves the parapharyngeal space; Type 3b is an extended sublingual ranula that extends across the parapharyngeal space, masticatory space, and/or the infratemporal fossa [6].

A ranula is a translucent or bluish cystic swelling on the floor of the mouth. Complications include interference with articulation, mastication, swallowing, infection, rupture, and, in severe cases (40-50%), it may cause tongue fall-back, leading to obstructive sleep apnea (OSA), speech delay, swallowing problems, and recurrent infections in the oral cavity [6].

A systematic review by Lucas et al. states that lesions on the floor of the mouth in children encompass a wide array of possible diagnoses, including congenital ranula, teratoma, dermoid cyst, foregut duplication cyst, vascular and lymphatic malformations, and thyroglossal duct cyst. Clinical evaluation remains the cornerstone for diagnosis [7]. Mucoceles can occur in various locations in the mouth, while ranulas are confined to the floor of the mouth.

Imaging (ultrasonogram, CT, and MRI), though not routinely recommended, may aid in ruling out other differential diagnoses and identifying underlying etiologies such as calculi. In an ultrasound, a ranula appears as a cystic mass without any calcifications. MRI helps in assessing lesion extension and in surgical planning [7].

Chung et al. outlined 30 different treatment modalities for ranula management: resection of the sublingual gland (partial or complete resection), excision of the ranula alone or aspiration of its contents, administering sclerosants, transcervical approaches, and/or submandibular sialoadenectomy, marsupialization, micro-marsupialization, and carbon dioxide laser surgery [8]. Niccoli-Filho and Morosolli also suggested carbon dioxide laser surgery [9].

## Conclusions

This case underscores the critical importance of a thorough oral cavity examination in neonates immediately after birth. Early identification of anomalies and prompt intervention can significantly enhance feeding efficiency, promote adequate nutrition, and support healthy growth and development. Timely clinical action can prevent complications and reduce parental anxiety. Raising awareness among healthcare professionals is vital to ensure such assessments become a routine part of newborn evaluations. By integrating careful oral assessments into standard neonatal care, healthcare providers can contribute to improved outcomes and a better quality of life for affected infants, reinforcing the value of vigilance in early neonatal screening practices.
